# Multimodal imaging guided preclinical trials of vascular targeting in prostate cancer

**DOI:** 10.18632/oncotarget.4463

**Published:** 2015-07-02

**Authors:** James Kalmuk, Margaret Folaron, Julian Buchinger, Roberto Pili, Mukund Seshadri

**Affiliations:** ^1^ Department of Pharmacology and Therapeutics, Buffalo, NY, USA; ^2^ Department of Molecular and Cellular Biophysics and Biochemistry, Buffalo, NY, USA; ^3^ Department of Medicine, Roswell Park Cancer Institute, Buffalo, NY, USA; ^4^ Current address: SUNY Upstate Medical University, Syracuse, NY, USA; ^5^ Current address: University at Buffalo – School of Medicine and Biomedical Sciences, Buffalo, NY, USA

**Keywords:** multimodality imaging, vascular disrupting agents, angiogenesis, prostate cancer, androgen deprivation therapy

## Abstract

The high mortality rate associated with castration-resistant prostate cancer (CRPC) underscores the need for improving therapeutic options for this patient population. The purpose of this study was to examine the potential of vascular targeting in prostate cancer. Experimental studies were carried out in subcutaneous and orthotopic Myc-CaP prostate tumors implanted into male FVB mice to examine the efficacy of a novel microtubule targeted vascular disrupting agent (VDA), EPC2407 (Crolibulin™). A non-invasive multimodality imaging approach based on magnetic resonance imaging (MRI), bioluminescence imaging (BLI), and ultrasound (US) was utilized to guide preclinical trial design and monitor tumor response to therapy. Imaging results were correlated with histopathologic assessment, tumor growth and survival analysis. Contrast-enhanced MRI revealed potent antivascular activity of EPC2407 against subcutaneous and orthotopic Myc-CaP tumors. Longitudinal BLI of Myc-CaP tumors expressing luciferase under the androgen response element (Myc-CaP/ARE-luc) revealed changes in AR signaling and reduction in intratumoral delivery of luciferin substrate following castration suggestive of reduced blood flow. This reduction in blood flow was validated by US and MRI. Combination treatment resulted in sustained vascular suppression, inhibition of tumor regrowth and conferred a survival benefit in both models. These results demonstrate the therapeutic potential of vascular targeting in combination with androgen deprivation against prostate cancer.

## INTRODUCTION

Prostate cancer is the most common non-cutaneous malignancy and the second leading cause of cancer death in American males [1]. Of newly diagnosed prostate cancers, approximately 75% of patients present with potentially aggressive tumors that demand therapeutic intervention [2]. The treatment paradigm for prostate cancer is based on practitioner based risk-assessment and ranges from observation (‘active surveillance’) to a diverse range of options including radical prostatectomy, radiotherapy and androgen deprivation therapy (ADT) [2, 3]. Given that most newly diagnosed prostate cancers are androgen dependent, ADT *via* chemical or surgical means remains a critical component of prostate cancer care [4–6]. However, ADT is not curative and tumor adaptation to castrate levels of serum androgen leads to the generation of castration-resistant prostate cancer (CRPC) that is metastatic and lethal. A number of potential mechanisms including androgen receptor (AR) amplification, AR mutation, and wild-type AR activation secondary to intra-tumoral androgen synthesis have been implicated in the generation of CRPC [7–9]. Progression to the castration-resistant state has also been associated with changes in vascular morphology and increased angiogenesis [10, 11]. Prostate tumors that recurred after ADT have been reported to express elevated levels of pro-angiogenic growth factors as well as have increased microvessel density [10, 12, 13]. Several preclinical studies have highlighted a strong link between androgens and angiogenesis in prostate cancer [14–16]. ADT has been shown to suppress production of vascular endothelial growth factor (VEGF) in tumor tissues [14]. Anti-androgen therapy has been shown to reduce prostatic blood supply and the luminal area of capillaries in the rat ventral prostate [15]. Androgen withdrawal has also been shown to inhibit tumor growth by reducing microvessel density and VEGF expression in castration-resistant prostate tumors [16]. Consistent with these findings, selective obliteration of immature vessels, down regulation of VEGF mRNA expression and increased endothelial apoptosis have also been reported in radical prostatectomy specimens obtained from patients subjected to androgen ablation [17]. Collectively, these observations have provided the rationale for examining the potential of vascular targeted therapies against prostate cancer.

The goal of the present study was to examine the therapeutic efficacy of a novel microtubule targeted vascular disrupting agent (VDA), EPC2407 (Crolibulin™) against prostate cancer. EPC2407 is a chromene analog that binds to endothelial microtubules promoting mitotic spindle destabilization, inhibition of tubulin polymerization and eventual induction of endothelial cell apoptosis [18]. Subsequent vascular destabilization leads to a reduction in intra-tumoral blood flow and tumor necrosis. The agent has recently completed Phase I evaluation in patients with solid tumors including prostate cancer [19]. We have recently reported on the vascular disruptive activity of EPC2407 against human head and neck cancer xenografts [20].

In the present study, we employed a multimodality functional imaging approach using three complementary imaging methods - magnetic resonance imaging (MRI), ultrasound (US) and bioluminescence imaging (BLI) to examine the potential of VDA therapy in prostate cancer alone and in combination with ADT in subcutaneous and orthotopic Myc-CaP prostate tumors. It was our hypothesis that imaging-guided scheduling of vascular-targeted therapy in combination with ADT would lead to enhanced therapeutic efficacy against prostate cancer.

## RESULTS

### Antivascular activity of EPC2407 against prostate cancer

**Figure 1:**
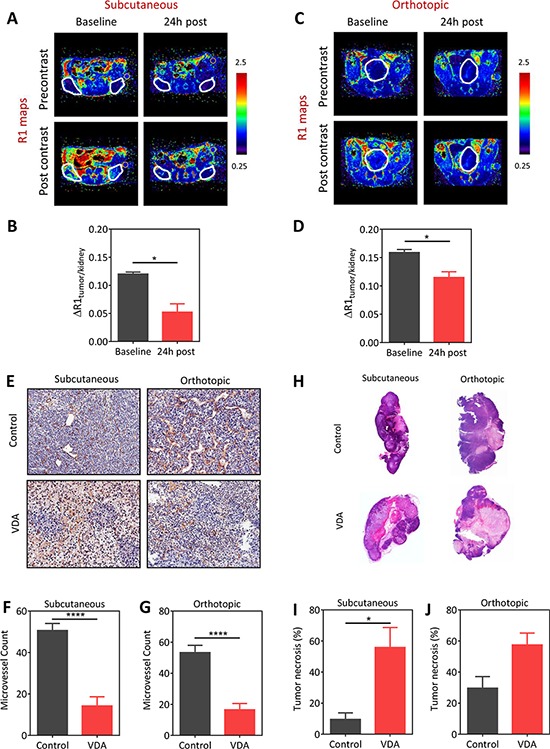
Vascular disruptive activity of EPC2407 against subcutaneous and orthotopic Myc-CaP prostate tumors evaluated by magnetic resonance imaging and immunohistochemistry **A–D**: Pseudo-colorized R1 (1/T1)-relaxation maps of mice bearing subcutaneous (A) and orthotopic (C) Myc-CaP tumors (*outlined in white*) at baseline and 24 hours post treatment with the VDA EPC2407. R1 maps were generated before (precontrast) and after (post contrast) administration of the MR contrast-enhancing agent, albumin-Gd-DTPA. Bar graphs show normalized R1 values of the tumor (ΔR1_tumor/kidney_) of subcutaneous (B; *n* = 4) and orthotopic (D; *n* = 3) tumors at the same time points. The kidney was chosen as a surrogate for contrast agent concentration in blood and to account for potential variations in contrast injection between the animals at different time points. A significant (*p* < 0.05) reduction in contrast agent uptake was seen in both models following VDA treatment. **E.** CD31-stained tumor sections from control and VDA-treated subcutaneous and orthotopic prostate tumors (*n* = 3–4/group) are shown. Corresponding microvessel counts (MVC) are shown in **F.** and **G.** A significant reduction in microvessel count (*p* < 0.001) was seen in VDA-treated tumors compared to control tumors in both models. **H.** Corresponding hematoxylin and eosin stained sections of control and VDA treated tumors in both models are shown. VDA-treated tumors showed increased necrosis compared to control tumors in both models. **I.** Subcutaneous Myc-CaP tumors showed a significant increase (*p* < 0.05) in tumor necrosis following VDA treatment compared to control tumors. **J.** Differences in tumor necrosis between control and VDA-treated tumors in orthotopic tumors was not statistically significant (*p* > 0.05). *denotes *p* < 0.05 ****p < 0.001.

We first examined the vascular disruptive activity of EPC2407 against prostate cancer using a combination of non-invasive MRI, immunohistochemistry (CD31), and histology (H&E). Contrast-enhanced MRI (CE-MRI) was performed before and 24 hours after administration of EPC2407 (20 mg/kg, i.v.) to assess changes in tumor perfusion following VDA treatment in subcutaneous (Figure 1A, 1B) and orthotopic (Figure 1C, 1D) Myc-CaP tumors. The panel of images shown in Figure 1A (subcutaneous) and Figure 1C (orthotopic) represent pseudo colorized R1 (1/T1)-relaxation maps of mice bearing Myc-CaP tumors (outlined in white) at baseline and 24 hours post VDA treatment. Images were obtained before (pre contrast) and after (post contrast) administration of the MR contrast agent, albumin-Gd-DTPA. A visible decrease in contrast enhancement was observed in both models following VDA treatment. Quantification of the contrast agent concentration confirmed a significant reduction (*p* < 0.05) in blood flow at the 24 hour time point in both subcutaneous (Figure 1B; *n* = 4) and orthotopic (Figure 1D; *n* = 3) tumors.

To validate our MRI findings, tumor sections obtained from control or VDA-treated tumors (*n* = 3–4/group) were immunostained for the endothelial cell marker, CD31. As shown in Figure 1E (*top panel*), control tumors at subcutaneous and orthotopic showed distinct CD31+ endothelial cell clusters with visible lumen. In comparison, VDA-treated tumors showed loss of CD31 staining in multiple regions within the tumor (Figure 1E, *bottom panel*). Quantification of microvessel counts revealed a significant reduction in VDA-treated tumors (*p* < 0.001) compared to controls (Figure 1F and 1G). Histologic evaluation showed increased necrosis in VDA-treated tumors compared to control tumors in both models (Figure 1H). Quantification of tumor necrosis (relative to whole tumor area) showed a significant increase (*p* < 0.05) following VDA treatment compared to control tumors in the subcutaneous model (Figure 1I). Differences in tumor necrosis between control and VDA-treated groups in orthotopic tumors was not statistically significant (*p* = 0.05; Figure 1J).

### Bioluminescence imaging of Myc-CaP/ARE-luc tumor response to castration

**Figure 2:**
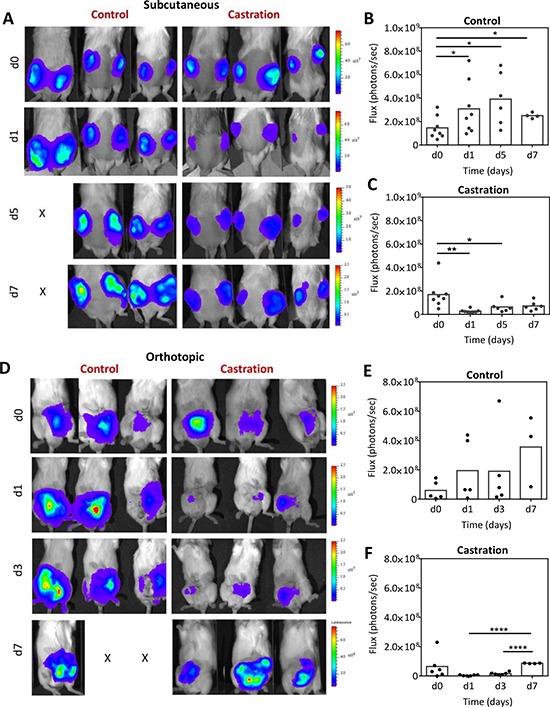
Bioluminescence imaging of Myc-CaP/ARE-luc tumor response to castration **A.** Longitudinal BLI of mice bearing subcutaneous Myc-CaP/ARE-luc tumors before and after castration. Corresponding photon flux values of tumors in intact controls **B.** and castrated **C.** animals (*n* = 8 per group) are also shown. BLI revealed a significant reduction in AR activity following castration (24 h) which was followed by a recovery over a 5–7 day period post castration. **D–F.** Longitudinal BLI of mice bearing orthotopic Myc-CaP/ARE-luc tumors in the control (*n* = 5) and castration (*n* = 6) arms at baseline (d0; before castration) and on days 1, 3 and 7 post castration. AR signal levels returned to baseline levels in orthotopic tumors on d7 post castration. *denotes *p* < 0.05, **denotes *p* < 0.01, ****denotes *p* < 0.001.

We performed BLI of subcutaneous and orthotopic Myc-CaP tumors transfected with firefly luciferase under the androgen response element promoter (Myc-CaP/ARE-luc). Longitudinal BLI was performed in independent cohorts of mice bearing bilateral subcutaneous (*n* = 8 per group) or orthotopic (*n* = 5–6 per group) Myc-CaP/ARE-luc tumors at baseline (d0; before castration) and at different times post castration (Figure 2). Figure 2A shows serial bioluminescence images of three mice bearing s.c. Myc-CaP tumors in the control (intact) and castration arms. Values of photon flux (reflective of AR signaling since luciferase expression is driven by the androgen response element) for the control and castration arms are also shown (Figure 2B). Control tumors showed increased bioluminescence flux over a 7 day period relative to baseline values (*p* < 0.05) reflective of unimpeded tumor growth. Lower flux values in the control arm on d7 potentially reflect the presence of large tumors with underlying necrosis. In comparison, a significant reduction (*p* < 0.01) in photon flux was observed at 24 h post castration. This reduction in flux was followed by a recovery in BLI signal over a 5–7 day period. This transient reduction and subsequent recovery of flux is reflective of changes in AR signaling following castration (Figure 2C). Similarly, in the orthotopic model (Figure 2D), control mice bearing orthotopic Myc-CaP/ARE-luc tumors showed increased photon flux over the 7 day period. Fluctuations in flux occurred with the loss of study subjects in the control arm at the 1 week time point (Figure 2D, 2E). Castration resulted in a significant loss of flux at 24 hours (Figure 2F). This reduction in BLI signal was transient with mean flux values returning to baseline levels within 1 week suggestive of the onset of castration-resistant disease.

### Multimodal imaging of Myc-CaP vascular response to castration

**Figure 3:**
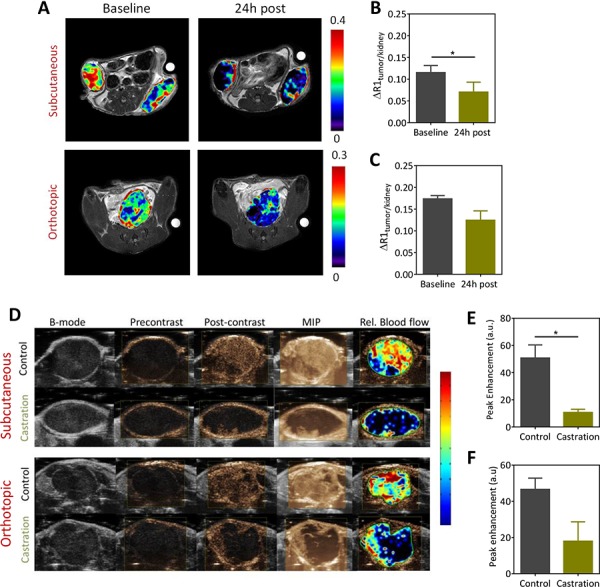
Contrast-enhanced MRI (CE-MRI) and contrast-enhanced ultrasound (CE-US) of Myc-CaP tumor vascular response to castration **A.** Pseudocolorized post contrast R1 (1/T1)-relaxation maps of mice bearing subcutaneous (*upper panel*) and orthotopic (*lower panel*) Myc-CaP/ARE-luc tumors overlaid onto the raw T2W proton images at baseline and 24 h post castration. Corresponding normalized values of the change in T1-relaxation rate for subcutaneous **B.** and orthotopic **C.** are shown. CE-MRI using an intravascular contrast agent, albumin-GdDTPA was performed in subcutaneous (*n* = 4) and orthotopic tumors (*n* = 3). A significant reduction in perfusion of subcutaneous Myc-CaP tumors was seen at the 24 h time point compared to baseline (*p* < 0.05). Difference between baseline and 24 h post treatment values in orthotopic tumors was not significant. **D.** CE-US images of subcutaneous (*upper panel*) and orthotopic (*lower panel*) Myc-CaP tumor vascular response to castration. (D) From left to right, the panel of images represent standard gray-scale (B-mode) US image, a contrast mode image prior to contrast injection (precontrast) and following contrast injection once a plateau in the signal intensity-time curve was achieved (post contrast), a maximum intensity projection (MIP) based on contrast accumulation (taken from the same frame), and a pseudocolorized map of the relative blood flow map for a tumor in each experimental group. Bar graphs showing peak enhancement (PE) values in subcutaneous **E.** and orthotopic **F.** Myc-CaP tumors calculated from the signal intensity-time curves in control and castrated animals. A significant reduction in PE was seen in subcutaneous Myc-CaP tumors following castraton. * denotes *p* < 0.05.

Given the androgen-angiogenesis link in prostate cancer, we examined the vascular response of Myc-CaP tumors to castration using CE-MRI and contrast-enhanced ultrasound (CE-US) (Figure 3). The panel of images shown in Figure 3A represents pseudo colorized contrast enhancement maps (ΔR1 maps) of mice bearing subcutaneous and orthotopic Myc-CaP tumors before and 24 hours post castration. A marked reduction in contrast enhancement was visible 24 hours post castration in subcutaneous Myc-CaP tumors (Figure 3A, *upper panel*). Normalized values of the change in R1 (1/T1)-relaxation rate (ΔR1_tumor/kidney_) also showed a significant reduction in contrast agent (Gd) concentration in tumors at this time point compared to baseline (Figure 3B; *p* < 0.05). Reduction in contrast enhancement (Figure 3A, *lower panel*) was also seen post castration in orthotopic tumors (Figure 3C). However, this reduction was not statistically significant (*p* > 0.05). We also performed CE-US examination in an independent set of animals bearing subcutaneous or orthotopic Myc-CaP tumors (Figure 3D–3F). The panel of images shown in Figure 3D represent standard gray-scale (B-mode) US image, a contrast mode image immediately prior to contrast injection (pre contrast) and following contrast injection once a plateau in the signal intensity-time curve was achieved (post contrast), a maximum intensity projection (MIP) based on contrast accumulation (taken from the same frame as the post-contrast image), and a pseudo colorized image of the relative blood flow within the tumor overlaid onto the B-mode image for a tumor in each experimental group. In the subcutaneous tumor model, a significant reduction in intratumoral delivery of the US contrast agent (peak enhancement) was seen in castrated mice at the 24 h time point compared to well-perfused untreated control tumors (Figure 3D, 3E). In comparison, the reduction in perfusion following castration in the orthotopic model was modest (Figure 3F; *p* > 0.05). We also performed dynamic BLI (dBLI) studies to assess the vascular response of Myc-CaP tumors to castration (Figure S1). These studies also revealed an acute but transient reduction in luciferin delivery 24 h post castration which was followed by vascular recovery approximately 5–7 days post castration.

### Vascular targeting in combination with castration against subcutaneous Myc-CaP tumors

**Figure 4:**
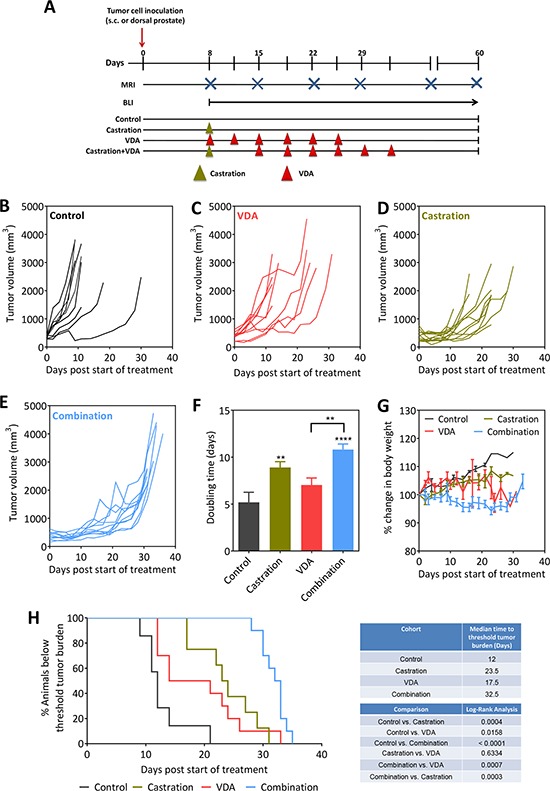
Efficacy of androgen deprivation in combination with vascular targeting against subcutaneous Myc-CaP tumors **A.** Schematic of experimental design. Tumor-bearing mice were randomized into control, castration, VDA, and castration + VDA groups. Caliper measurements were obtained in mice bearing subcutaneous tumors while bioluminescence imaging along with MRI was performed to monitor growth of orthotopic tumors. **B–E.** Growth curves of s.c. Myc-CaP tumors in control, castration alone, VDA alone and combination arms. Tumor volumes were calculated from caliper measurements as described in Materials and Methods. Tumor doubling times **F.** and change in body weight **G.** of animals in all 4 experimental groups are shown. **H.** Kaplan-Meier survival curves of animals bearing subcutaneous Myc-CaP tumors in control and treatment groups. Median survival (days) based on percentage of animals below threshold tumor burden (based on ethical guidelines) and log-rank test *p*-values for each cohort compared to untreated control and between groups are shown. **p < 0.01 ****p < 0.001.

Next, we examined if targeting the vascular recovery post castration using EPC2407 would enhance the therapeutic efficacy of ADT *in vivo*. The study design and the experimental groups are shown schematically in Figure 4A. Mice bearing subcutaneous Myc-CaP tumors were randomized into one of 4 experimental arms: untreated control (*n* = 9), castration alone (*n* = 10), EPC2407 alone (*n* = 10), and, combination (*n* = 10). For combination treatment, the VDA EPC2407 was administered at a dose of 20 mg/kg (i.v.) twice a week for three weeks beginning at 7 days post castration. Individual tumor growth curves of animals in all 4 groups based on tumor volumes (calculated from caliper measurements) are shown in Figure 4B–4E. As monotherapies, castration and EPC2407 resulted in marginal delay in tumor growth compared to untreated control tumors. Tumor doubling times (dT) calculated from growth curves showed a significant increase in dT following castration alone while VDA treatment alone did not result in any significant change in dT. Animals treated with the combination showed the greatest increase in tumor dT (Figure 4F). Body weight measurements were recorded as a read-out of treatment related toxicity. Animals treated with combination treatment showed minimal loss of body weight (<5%) during the duration of treatment without any evidence of sustained long-term toxicity (Figure 4G). Kaplan-Meier analysis revealed significant improvement in median survival (based on time to reach threshold tumor burden) of animals treated with combination compared to controls and either monotherapy (Figure 4H).

### VDA treatment effectively suppresses tumor vascular recovery following castration and confers survival benefit in orthotopic Myc-CaP tumor model

**Figure 5:**
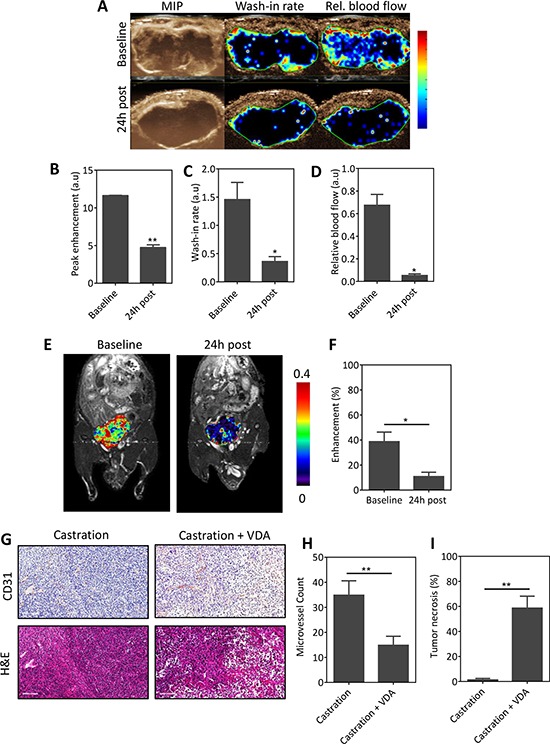
VDA treatment inhibits vascular recovery following castration **A.** CE-US of vascular response of orthotopic Myc-CaP tumors (*n* = 3) to combination treatment. From left to right, images represent a maximum intensity projection (MIP), and parametric maps of wash-in rate and relative blood flow of an orthotopic Myc-CaP tumor at baseline (d7 post castration) and 24 h post VDA treatment (d8). Bar graphs showing quantitative estimates of perfusion values in arbitrary units (A.U.): peak enhancement **B.** wash-in rate **C.** and relative blood flow **D.** derived from the contrast signal intensity-time curves. A significant reduction in multiple parameters of vascular function was seen following combination treatment. **E.** and **F.** 3D MR angiography of orthotopic Myc-CaP response to combination treatment. (E) Pseudo colorized enhancement maps of signal intensity in a mouse bearing orthotopic Myc-CaP tumors at baseline (d7 post castration) and 24 h post VDA treatment overlaid on raw proton images. Consistent with the CE-US data, a significant reduction in enhancement was seen 24 h post VDA treatment indicative of a reduction in tumor blood flow (contrast agent delivery). **G.** Photomicrographs of CD31 and H&E sections of orthotopic Myc-CaP tumors (*n* = 3–4 per group) excised following castration and combination treatment (castration + VDA). Combination treatment resulted in a significant reduction in microvessel density **H.** which was associated with increased tumor necrosis **I.** compared to castration alone. *denotes *p* < 0.05, **denotes *p* < 0.01.

To determine if VDA treatment could inhibit the vascular recovery following castration, mice bearing orthotopic Myc-CaP tumors were administered the VDA EPC2407 (20 mg/kg, i.v.) 1 week post castration. CE-US and CE-MRI examination were performed at baseline (d7 post castration) and 24 h post VDA treatment (d8). The panel of images shown in Figure 5A includes a MIP image and parametric maps of wash-in rate and relative blood flow of an orthotopic Myc-CaP tumor at the two time points. In support of our hypothesis, treatment with the VDA in combination with castration resulted in a significant reduction in multiple vascular parameters on CE-US examination (Figure 5B–5D). Three-dimensional (3D) MR angiography (Figure 5E) showed evidence of reduced perfusion following VDA treatment in castrated mice (Figure 5F). Immunostaining of tumor sections for the endothelial cell marker confirmed tumor vascular damage and necrosis following combination treatment compared to castration alone (Figure 5G). Quantitative estimates of vascularity from CD31-immunostained tumor sections also showed a significant reduction in microvessel counts (Figure 5H) in mice treated with combination (14 ± 3, 24 fields, *p* < 0.01) compared to castration alone (34 ± 5, 25 fields). Quantitative analysis of H&E stained sections also showed a significant (*p* < 0.01) increase in tumor necrosis following combination treatment (Figure 5I).

**Figure 6:**
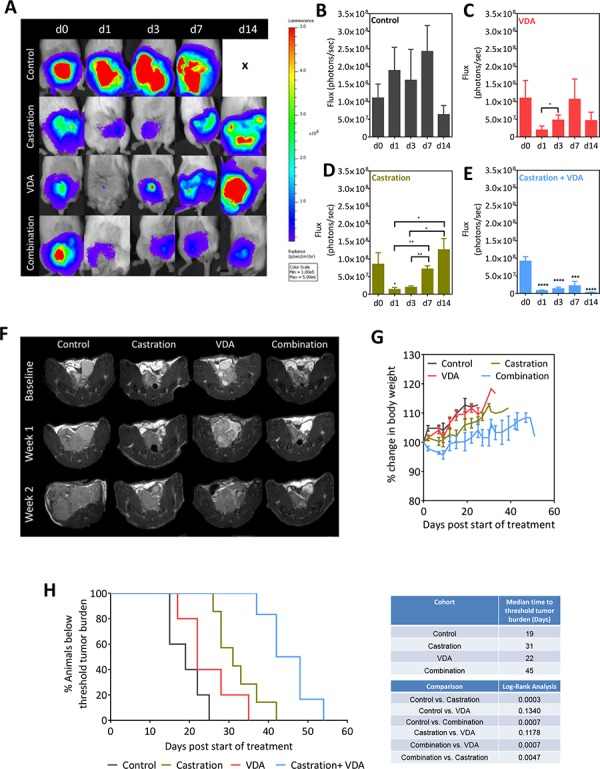
BLI and MRI based monitoring of therapeutic efficacy of EPC2407 in combination with castration against orthotopic Myc-CaP tumors **A.** Bioluminescence images of mice bearing orthotopic luciferase-transfected Myc-CaP tumors and treated with castration alone, VDA alone or combination. **B–E.** Photon flux values over time for control (*n* = 7), castration alone (*n* = 6), VDA alone (*n* = 8) and combination arms (*n* = 10). Combination treatment resulted in sustained inhibition of tumor growth as evidenced from the low flux levels. **F.** Axial T2-weighted MR images of mice bearing orthotopic Myc-CaP tumors from all 4 experimental arms. Compared to either monotherapy, combination treatment resulted in sustained inhibition of tumor growth and delayed the onset of castration recurrent disease. **G.** No significant difference in body weight was observed between the 4 groups. **H.** Kaplan-Meier survival curves of animals bearing orthotopic Myc-CaP tumors in control and treatment groups. Median survival (days) of animals calculated based on time to reach threshold tumor burden for euthanasia and log-rank test *p*-values for each cohort compared to untreated control and between groups are shown. Combination treatment conferred a statistically significant survival benefit in this model with an improved median survival compared to castration alone and VDA alone. *denotes *p* < 0.05, **denotes *p* < 0.01, *** denotes *p* < 0.005, ****denotes *p* < 0.001.

To determine if inhibiting the vascular recovery post castration using EPC2407 translated into long-term therapeutic benefit, mice bearing intra-prostatic Myc-CaP tumors were randomized into untreated control (*n* = 7), castration alone (*n* = 6), single agent VDA (*n* = 8) or combination arms (*n* = 10) using the study design shown in Figure 4A. Animals underwent BLI (Figure 6A–6E) and MRI (Figure 6F) examination over a 2-week period. As previously noted, in untreated control mice there was consistent elevation in tumor flux until the 2 week time-point, at which point loss of study subjects contributed to the reduction in mean flux values (Figure 6B). In castrated mice flux values were transiently reduced over the first week but subsequently exceeded baseline values in 2 weeks indicative of castration recurrent disease (Figure 6D). While single agent VDA treatment had minimal effect on tumor growth (Figure 6C), combination treatment resulted in sustained inhibition of tumor growth as shown in Figure 6E. In addition to BLI, T2-weighted MRI examination was performed once a week to monitor tumor growth *in vivo* (*n* = 5–7 per group). Figure 6F shows axial T2-weighted MR images of mice bearing orthotopic Myc-CaP tumors from all 4 experimental arms. The individual tumor volumes of animals are shown in Supplementary Figure S2. As shown in Figure 6F, compared to either monotherapy, combination treatment resulted in sustained inhibition of tumor growth and delayed the onset of castration recurrent disease. No significant difference in body weight was observed between the 4 groups (Figure 6G). Sustained tumor growth inhibition following combination treatment conferred a statistically significant survival benefit (Figure 6H; *p* < 0.001) in this model with an improved median survival of 45 days compared to castration alone (31 days) and VDA alone (22 days).

## DISCUSSION

Angiogenesis is a necessity for perpetuation of neoplastic growth [21]. Prostate cancer is no exception [10–16]. Multiple classes of vascular-targeted therapeutics ranging from vascular endothelial growth factor inhibitors [22–24], tyrosine kinase inhibitors [25, 26] and immunomodulatory agents such as linomide [27], thalidomide [28], and tasquinimod [29] have been evaluated for their antiangiogenic and antitumor activity against prostate cancer with mixed results. Regulatory approval of some of these novel agents has enhanced the number of treatment options for patients while simultaneously raising questions regarding the timing, therapeutic sequence and duration of such therapies, particularly in combination with ADT [30]. Given the anti-vascular effects of ADT, determining the optimal timing and sequence becomes even more critical when ADT is combined with other agents that suppress the same target (i.e. blood vessels). However, most clinical trials are aimed at conducting ‘head-to-head’ comparisons between therapies to determine superiority [30]. Considering the intellectual and financial challenges associated with conducting such optimization trials in the clinic, it would be wise to conduct such optimization studies in preclinical models to better inform future clinical trial design [31]. In this regard, non-invasive imaging methods can enable determination of the kinetics of tumor response or regrowth following ADT and potentially guide the study design of combinatorial trials. Functional imaging methods can provide read-outs of early vascular changes following ADT within the prostate microenvironment and provide insight into mechanisms of disease recurrence. Informed scheduling from imaging-guided preclinical trials may therefore help identify a therapeutic window during which vascular-targeted agents can be safely and effectively administered in combination with ADT.

In the present study, we employed a multimodality functional imaging approach using three complementary imaging methods, namely, MRI, US and BLI to evaluate the therapeutic potential of VDA therapy in combination with ADT against prostate cancer. The high sensitivity and rapid data acquisition afforded by BLI enables longitudinal assessment of tumor burden in a high-throughput manner [32, 33]. However, changes in BLI flux could be related to changes in cell number/viability, altered delivery of luciferin substrate to the tumor, and changes in transcription through the promoter (ARE in our study). To determine if the reduced BLI signal was related to impaired luciferin delivery, we performed dBLI acquisition of tumor-bearing animals at different time points following castration. Published observations by us and others have shown that dBLI can be successfully utilized to assess tumor vascular integrity and response to vascular-targeted therapeutics [34–37]. Our dBLI studies provided evidence of tumor vascular recovery approximately 5–7 days post castration. This is consistent with a previous study in short-term human prostate tissue xenografts by Godoy *et al.* [38] in which castration resulted in an acute reduction in microvessel density and elevations in VEGF expression. However, this anti-vascular effect of ADT was transient and was followed by vascular recovery between 7–14 days post castration [38]. In our study, imaging studies helped determine the kinetics of vascular remodeling and tumor regrowth following castration and guide preclinical trial design. Administration of VDA one-week post castration inhibited vascular recovery post castration and led to a significant survival benefit compared to either monotherapy. Collectively, the results of our studies demonstrate the usefulness of functional imaging in guiding study design and assessment of tumor response to vascular-targeted combination strategies.

While BLI is extensively used in preclinical research, it is not directly applicable to clinical medicine. Therefore, to validate our dBLI observations, we utilized CE-MRI and CE-US, two clinically utilized imaging modalities that allow for morphologic and functional vascular assessment of tumors without the use of ionizing radiation. Consistent with our dBLI observation, CE-MRI showed reduction in perfusion following castration which was further enhanced by EPC2407. CE-MRI methods have been widely used in the diagnostic and therapeutic assessment of prostate cancer [39, 40]. Decreases in vascular permeability and tumor volume have also been reported on MRI examination of patients with biopsy proven prostate cancer following ADT [41]. Similarly, the value of ultrasound methods including CE-US and Doppler imaging in the assessment of prostate cancer has been demonstrated in preclinical [42] and clinical studies [43, 44]. We have previously utilized CE-US to monitor tumor response to vascular-targeted therapies in experimental tumor models [20, 45]. Our dBLI results suggest that vascular recovery post castration occurs 5–7 days post treatment in this model. Importantly, these vascular changes detected by dBLI preceded any change in tumor volume following castration (Figure S3) which was observed ∼18–19 days post castration in the subcutaneous model) and ∼15 days post castration in orthotopic Myc-CaP tumors. Similarly, Okihara *et al*., have shown that reduction in vascular flow precedes change in prostate volume following castration in patients [43]. Collectively, these observations highlight the significance of the androgen-angiogenesis axis in prostate cancer. Our results also demonstrate that non-invasive multimodal imaging approaches can provide useful insight into the molecular and functional changes occurring in the tumor microenvironment and guide the design of combination therapies.

The two principal approaches to tumor vascular targeting include (i) inhibition of new vessel growth with anti-angiogenic agents [46] and, (ii) destruction of existing vasculature with VDAs [47]. Tumor blood vessels themselves appear to be susceptible to the action of VDAs, potentially due to a combination of their inherently chaotic nature and poor framework of pericytes and endothelial cells [47]. To date, evaluation of the therapeutic potential of vascular-targeted agents against prostate cancer has focused primarily on inhibition of the VEGF pathway with anti-angiogenic agents. Clinical trials seeking to inhibit the VEGF pathway with the anti-angiogenic monoclonal antibody, Bevacizumab, have been disappointing in both single agent and combination settings [22, 23], while the recently completed VENICE trial combining the VEGF inhibitor, Aflibercept, with Docetaxel and Prednisone reported increased rates of grade 3–4 toxicity with no improvement in survival [24]. In comparison, a Phase II study by Pili *et al*., showed that the addition of the VDA ASA404 to Docetaxel in metastatic CRPC was shown to have acceptable toxicity and may improve efficacy over Docetaxel alone [48]. Further clinical development of ASA404 was halted following the failure of the agent in a Phase III trial against non-small cell lung cancer [49]. Nevertheless, several VDAs are currently undergoing Phase I-II clinical evaluation in patients with advanced solid tumors [50, 51], highlighting the sustained interest in this therapeutic approach. The differential vascular response of subcutaneous and orthotopic tumors to EPC2407 observed in our studies highlights the influence of the microenvironment on tumor angiogenesis and the need for evaluating the activity of this class of agents in clinically-relevant, orthotopic tumor models.

The intrinsic relationship between androgens and angiogenesis in the biology of prostate cancer [10–16, 52] supports the utilization of anti-vascular therapy against this disease. However, the utilization of vascular-targeted agents against prostate cancer in the post castration setting creates therapeutic opportunities and challenges. Clinical studies have reported elevated levels of hypoxia exist within 30–90% of prostate tumors [53]. In preclinical studies of prostate xenografts, hypoxia has been shown to promote hypoxia-Inducible factor-1 α (HIF-1 α) and VEGF up regulation, increased cellular proliferation, decreased apoptosis, down regulation of DNA repair proteins, as well as radioresistance and chemoresistance [53]. Given the marked reduction in tumor perfusion post treatment, the use of VDAs in prostate cancer may raise valid concerns about potential worsening of tumor hypoxia and subsequent promotion of an aggressive phenotype. However, in our study, we did not observe any evidence to support this concern. Nevertheless, to eliminate this possibility, one could target the hypoxic regions within the tumor using bio-reductive drugs such as tirapazamine [54] or AQ4N [55]. In a rat tumor model, Johansson *et al*. have previously reported that castration transiently increases hypoxia, and subsequent administration of Tirapazamine can improve the therapeutic efficacy of castration [56]. More recently, Ming *et al*., have shown that informed scheduling of AQ4N increases the effectiveness of bicalutamide treatment against LNCaP tumors [55].

We recognize the limitations of our study. While our imaging-guided treatment schedule translated into encouraging survival benefit *in vivo*, the lack of durable responses or ‘cure’ in our studies suggests that further optimization of the timing of intervention and duration of treatment is needed. In our study, multiple tumors in the combination group did not show any marked change in tumor volume until after cessation of VDA therapy, suggesting that VDA was indeed inhibiting the angiogenic rebound following castration critical for development of CRPC. This also suggests that long-term VDA therapy may be beneficial for sustained inhibition of CRPC. Future studies should therefore examine the efficacy of this combination treatment against early vs. advanced disease and evaluate alternative schedules (e.g. continuous vs. intermittent dosing). However, adequate consideration should therefore be given to the toxicity profiles of VDAs while designing such preclinical trials. The VDA dose (20 mg/kg, i.v.) used in our study was identified based on echocardiographic measurements performed in our laboratory that showed no evidence of cardiotoxicity in mice. Importantly but perhaps not surprisingly, we observed significant toxicity (50% mortality) when the VDA was administered on the day of surgical castration. While this could be attributed to the considerable stress associated with treatment (anesthesia, surgical procedure etc.), the observations highlight the impact of schedule and sequence on safety and efficacy. Second, all of our experimental studies were conducted in the Myc-CaP tumor model. Although the model recapitulates the molecular profile of human disease, it would be important to examine the activity of combination treatment in transgenic models or patient-derived xenograft models of prostate cancer to fully realize the therapeutic potential of this approach. And finally, given the primary cause of mortality in prostate cancer patients is often metastatic spread, it would also be important to examine the potential of VDA therapy in the metastatic setting. We have begun answering some of these questions in our laboratory using metastatic tumor models and will report on our findings in the future.

## MATERIALS AND METHODS

### Tumor model system

The murine prostate cancer cell line Myc-CaP [57], a generous gift from Dr. Charles Sawyers, was stably transfected with firefly luciferase under the androgen response element promoter (Myc-CaP/ARE-luc) as previously described [58]. Cells were maintained in complete media (complete Dulbecco's Modified Eagle's medium, 10% fetal bovine serum and 5% penicillin/streptomycin) in cell culture flasks. All cells were incubated at 5% CO_2_. Tumors were established in 8–12 week old wild-type male FVB mice (NCI Frederick, MD, USA). Animals received food and water *ad libitum* and were maintained on a 12-hour light/dark cycle within a HEPA-filtered environment. Tumors were established *via* inoculation of Myc-CaP cells (∼5 × 10^5^ suspended in 50 μL of Matrigel) into the flanks (subcutaneous model) or dorsal prostate (orthotopic model). Orthotopic implantation began with removal of abdominal hair followed by formation of a 1.5 cm incision 5–8mm above the genitalia. The bladder and seminal vesicles were gently extruded with cotton swabs and cells were injected into the dorsal prostate. The wound was then sutured with 2–0 Vicryl absorbable sutures. All experimental procedures utilized aseptic technique and were in accordance with approved protocols by the Institutional Animal Care and Use Committee (IACUC) at Roswell Park Cancer Institute.

### Treatments

EPC2407 (Crolibulin™): The agent (kindly provided by Epicept Corporation, Tarrytown, USA) was dissolved in manufacturer's recommended vehicle consisting of phosphate buffered saline, polyethylene glycol, and lutrol (to enhance solubility of EPC2407) (Sigma-Aldrich, St. Louis, MO) at a concentration of 5 mg/ml and was administered at a dose of 20 mg/kg via intravenous (i.v.) injection twice a week for three weeks.

Castration: A cranial-caudal incision was made in the scrotal sac following depilation with NAIR (Church & Dwight Co., Inc., Ewing, NJ). Gentle abdominal pressure allowed for extrusion of the testes. Mosquito clamps were used to grasp the epididymis, while the connective fascia was dissected away. The testicles were then excised and the incision wound was sutured with 2–0 Vicryl absorbable sutures.

### Bioluminescence imaging

Dynamic bioluminescence imaging (dBLI) [36, 37] was performed using the Xenogen IVIS Spectrum imaging system (Caliper Life Sciences, Alameda, CA). D-Luciferin (Gold Biotechnologies, St. Louis, MO) was administered (75 mg/kg; s.c.) 2 minutes prior to imaging and mice were serial imaged for 10 minutes at 1 minute intervals. The imaging parameters for all acquisitions were: medium binning, 1 f/stop, a blocked excitation filter and an open emission filter, and a 22 cm field of view to image 3 mice at once. Image post-processing and signal intensity quantification (reported as flux, or photons per second) was carried out using Living Image software (Caliper Life Sciences, Alameda, CA).

### Ultrasound

Two-dimensional brightness-mode (B-mode) images were acquired utilizing the small animal, high-resolution imaging system (Vevo 2100; Visualsonics Corporation, Toronto, Canada) as described previously [20, 45]. Briefly, tumor-bearing mice were anesthetized with Isoflurane (Benson Medical Industries, Markham, ON, Canada) and positioned on a heated platform (THM 150; Indus Instruments, Webster, TX) equipped with integrated temperature sensor and ECG electrodes for imaging. All four paws were secured onto the ECG pads and body temperature was kept between 34 and 37°C. Following depilation, an ultrasound acoustic gel (Aquasonic 100, Parker Laboratories Inc., Fairfield, New Jersey) was applied to facilitate ultrasound transmission from the transducer to the skin. A solid state transducer (MS-250SC) was placed on the tumor and held in position by a clamp mounted on the Vevo Rail System. This transducer has a center frequency of 18 MHz and an axial resolution of 75 μm at an elevation of 8 mm. Nonlinear Contrast Mode imaging (32) was employed to detect the presence of Vevo MicroMarker contrast agent (VisualSonics, Toronto, ON, Canada). A bolus injection of 50 μl (concentration of 2 × 10^9^ microbubbles per ml) was administered through a 25-gauge needle inserted into the tail vein. Acquisition parameters were kept constant at 4% power, 35 dB contrast gain, gate size of 6, high line density, and standard beam width. Post-processing and parametric map production was performed utilizing the VEVOCQ software utilizing regions of interest drawn around the tumor with perfusion parameters (peak enhancement, wash-in rate and relative blood flow) derived from intratumoral signal intensity-time curves (VisualSonics, Toronto, ON, Canada) similar to methods previously described by Rissanen et al [45, 59].

### Magnetic resonance imaging

Experimental MRI examinations were performed in a 4.7T/33-cm horizontal bore magnet (GE NMR Instruments, Fremont, CA) equipped with AVANCE digital electronics (Bruker Biospec, Paravision 3.0.2; Bruker Medical Inc., Billerica, MA), a removable gradient coil insert (G060) generating a maximum field strength of 950 mT/m and a custom-designed 35-mm RF transmit-receive coil. Animals were secured in a form-fitted, MR compatible sled (Dazai Research Instruments, Toronto, Canada) equipped with temperature and respiratory monitoring sensors. Animal body temperature was maintained at 37°C during imaging using an air heater system (SA Instruments Inc., Stony Brook, NY), and automatic temperature feedback was initiated through thermocouples in the sled. A phantom containing 0.15 mM gadopentetate dimeglumine (Gd-DTPA; Magnevist, Berlex Laboratories, Wayne, NJ) was used for monitoring changes in noise and system performance. Preliminary localizer images were acquired for subsequent slice prescription. Tumor volumes were calculated from multislice T2-weighted (T2W) spin echo images with the following parameters: Field of view (FOV) = 3.20 × 3.20 cm, matrix (MTX) = 256 × 192, slice thickness = 1 mm, NEX = 4, TR = 2500 ms, TE_eff_ = 41.0 ms, RARE/Echoes= 8/8. T1-relaxation rate measurements were obtained using a saturation recovery, fast spin echo (FSE) sequence with a TE_eff_ = 10 ms, TR ranging from 360 to 6000 ms, FOV= 3.20 × 3.20 cm, slice thickness = 1 mm, MTX = 128 × 96, NEX = 3, RARE = 8, acquisition time = 4m 50s. Three axial T1-FSE scans were acquired before contrast and five T1-FSE scans were acquired after administration of 0.05mmol/kg albumin-GdDTPA. Three-dimensional MR angiography was performed using a spoiled-gradient echo protocol as described previously [60]. Following image acquisition, raw image sets were transferred to a processing workstation and converted into Analyze™ format (AnalyzeDirect, version 7.0; Overland Park, KS). All post processing of imaging data was carried out in Analyze™ and MATLAB. A region of interest (ROI) was manually traced around the entire tumor area on each tumor slice. Tumor volume was calculated by measuring the cross sectional area on each slice and multiplying their sum by the slice thickness. R1 (1/T1)-relaxation maps were calculated on a pixel-by-pixel basis in MATLAB. For each treatment group, R1 maps were generated at baseline (pretreatment) and 24 hours post treatment by subtracting the mean post contrast R1map from the mean pre contrast R1 map of the same animal. Calculated values of tumor ΔR1 were normalized to the change in kidney R1 values post contrast (as a surrogate for concentration of the contrast agent in blood) to account for potential differences in contrast agent injection between time points. Normalized signal intensity values obtained from 3D angiographic images were normalized to the phantom to obtain relative intensity values (RI) and percent enhancement (E) calculated using the formula, E = [(RI_post contrast_ − RI_pre contrast_)/RI_pre contrast_] × 100 as previously described [60, 61].

### Immunohistochemistry and histology

Resected tumors were placed in zinc fixative (BD Biosciences, San Diego, CA) for CD31 immunostaining and histology (H&E). Paraffin sections were cut at 4 μm, placed on charged slides, and dried at 60°C for one hour. Slides were cooled to room temperature, deparaffinized in three changes of xylene, and rehydrated using graded alcohols. No antigen retrieval step was necessary because slides were zinc fixed. Endogenous peroxidase was quenched with aqueous 3% H_2_O_2_ for 10 minutes and washed with PBS/T. Slides were loaded on a DAKO autostainer and serum free protein block (DAKO, Carpinteria, CA) was applied for 5 minutes, blown off, and the antibodies were applied for one hour. The Pathology Core Facility at Roswell Park Cancer Institute supplied positive and negative control slides. Biotinylated goat anti-rat IgG (BD Pharmingen, Franklin Lakes, NJ) secondary antibody was applied, followed by Streptavadin HRP conjugate (Invitrogen, Grand Island, NY) for 30 minutes, and the DAB chromogen (Dako, Carpinteria, CA) for 5 minutes. For H&E staining, paraffin sections were cut at 4 μm, placed on charged slides, and dried at 60°C for one hour. Slides were cooled to room temperature, deparaffinized in three changes of xylene, and the tissue sections were carried through a series of rehydration steps from xylene to distilled water. Nuclei were stained with Harris Hematoxylin (Poly Scientific, Bay Shore, NY), rinsed in running tap water, and then differentiated with 0.3% acid alcohol (Sigma-Aldrich, St. Louis, MO). 0.2% Ammonium Hydroxide was used for bluing and slides were then transferred to 95% ethanol before Eosin-Y staining (Thermo Fisher Scientific, Fremont, CA). Slides were then dehydrated, cleared and cover slipped. To quantify the degree of necrosis in H&E samples, acquired 1X images were uploaded into Analyze software and specified regions of interest (ROI) were created to compare the overall tumor area and the regions of necrosis, which was then reported as percentage tumor necrosis [37]. A minimum of 3 tumors per group with 5 fields/tumor (20 × magnification) were evaluated for quantification of microvessel density. Images were digitized using the ScanScope XT system and ImageScope software (Aperio Technologies, Vista, CA).

### Therapeutic response assessment

For animals bearing subcutaneous Myc-CaP tumors, caliper measurements were recorded 2–3 times per week and were used to calculate tumor volume (mm^3^) using the formula, V = 1/2(L × W^2^), where L is the longest axis, and W is the axis perpendicular to L. Calculated volumes were plotted as a function of time. All treatments were initiated using experimental groups with matched tumor volumes. Orthotopic Myc-CaP tumor volumes were calculated from multi-slice T2W MRI acquired once a week. Animals were euthanized when tumor burden reached the threshold for euthanasia, sustained reduction in body weight (20% or greater from baseline measurements) or if moribund status was noted (minimal movement, labored breathing) as per institutional protocols.

### Statistical considerations

All statistical analyses were performed using Graphpad Prism (GraphPad Software, San Diego, CA). Mean ± standard error is reported for all datasets. *P*-values < 0.05 were considered significant. MRI based measurements on the same subjects between time points were assessed using a two-tailed paired *t* test. Differences in microvessel density and necrosis between control and treatment groups were analyzed using an unpaired *t* test. Comparisons between multiple groups or multiple time points were analyzed using a one-way analysis of variance (ANOVA). Tumor-doubling times were calculated from the change in tumor volume growth curve (consisting of volume measurements acquired every 2–3 days) using an exponential growth equation and differences between treatment groups were analyzed for statistical significance using a one-way ANOVA. Kaplan-Meier survival curves were generated based on time to reach threshold tumor burden and differences in median survival analyzed using the log-rank test.

## SUPPLEMENTARY FIGURES



## Abbreviations

MRI: Magnetic resonance imaging
US: Ultrasound
BLI: Bioluminescence imaging
CE-MRI: Contrast-enhanced magnetic resonance imaging
CE-US: Contrast-enhanced ultrasound
dBLI: Dynamic bioluminescence imaging
VDA: Vascular disrupting agent
ADT: Androgen deprivation therapy
CRPC: Castration resistant prostate cancer
