# Supplementary material for: Multimodal imaging guided preclinical trials of vascular targeting in prostate cancer
Source: Oncotarget. 2015 Jul 2;6(27):24376–92. doi: 10.18632/oncotarget.4463 (PMC4695192; doi:10.18632/oncotarget.4463)
Supplement: Supplementary file 1 [file oncotarget-06-24376-s001.pdf]

SUPPLEMENTARY FIGURES

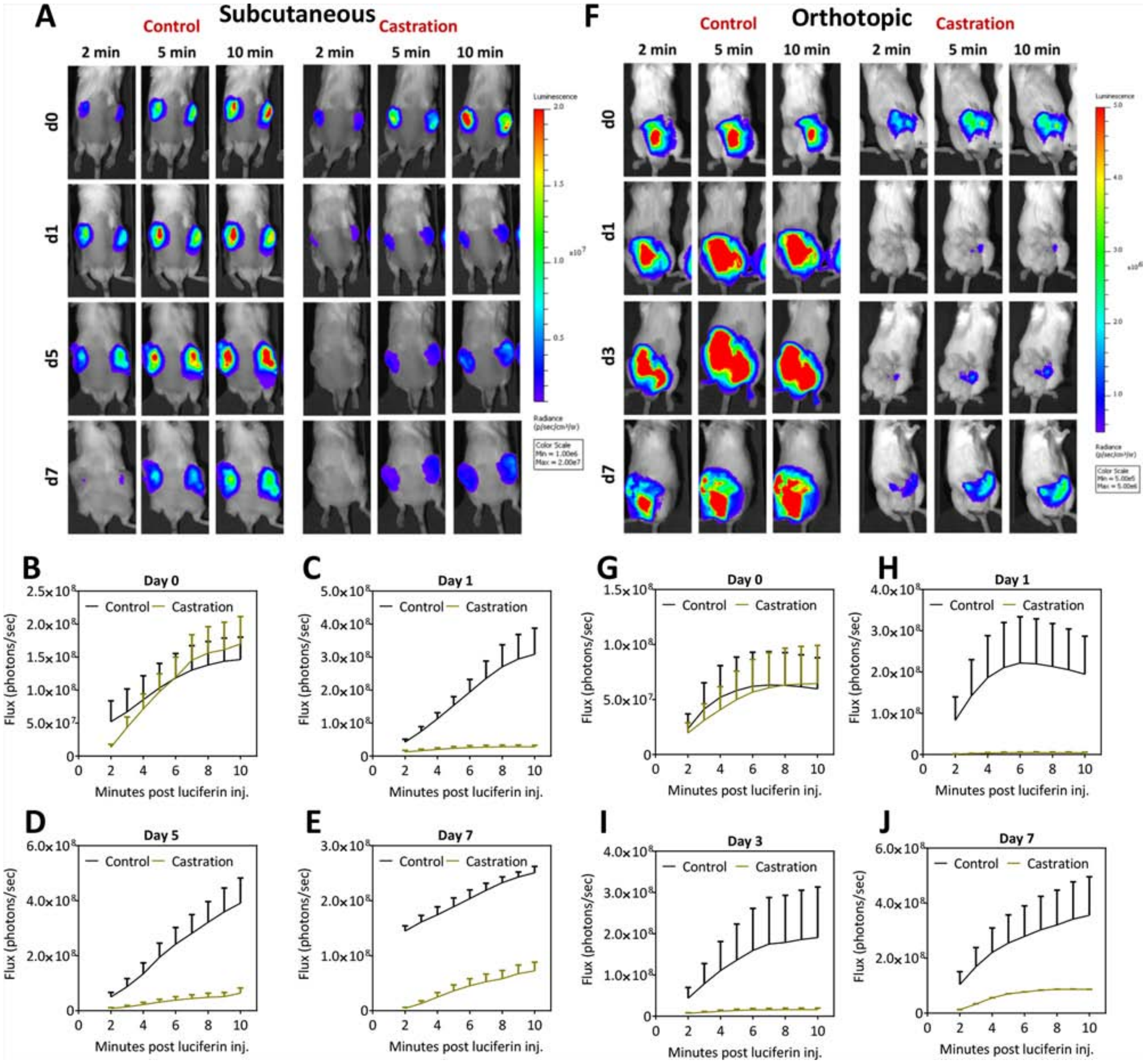

Supplementary Figure S1: Dynamic bioluminescence imaging (dBLI) of Myc-CaP vascular response to castration. Serial bioluminescence images of mice bearing subcutaneous (**A**; n = 8 per group) and orthotopic (**F**; n = 5–6 per group) Myc-CaP/ARE-luc tumors in intact controls and castrated animals on day 0 (pre-castration) and at different time points post castration. Corresponding curves of the kinetics of light emission (photon flux) at different times post luciferin injection are shown for subcutaneous **B–E.** and orthotopic tumors **G–J.** Temporal curves of BLI flux of control tumors showed comparable enhancement patterns (kinetics of photon flux) following luciferin injection at the different time points. In comparison, a marked reduction in photon flux was observed 24 hours post castration ($p < 0.01$) with no evidence of enhancement in flux over the 10 minutes post luciferin administration in both models (C, H). The reduction in BLI signal persisted for a few days with a gradual recovery observed on day 7 post castration (E, J).

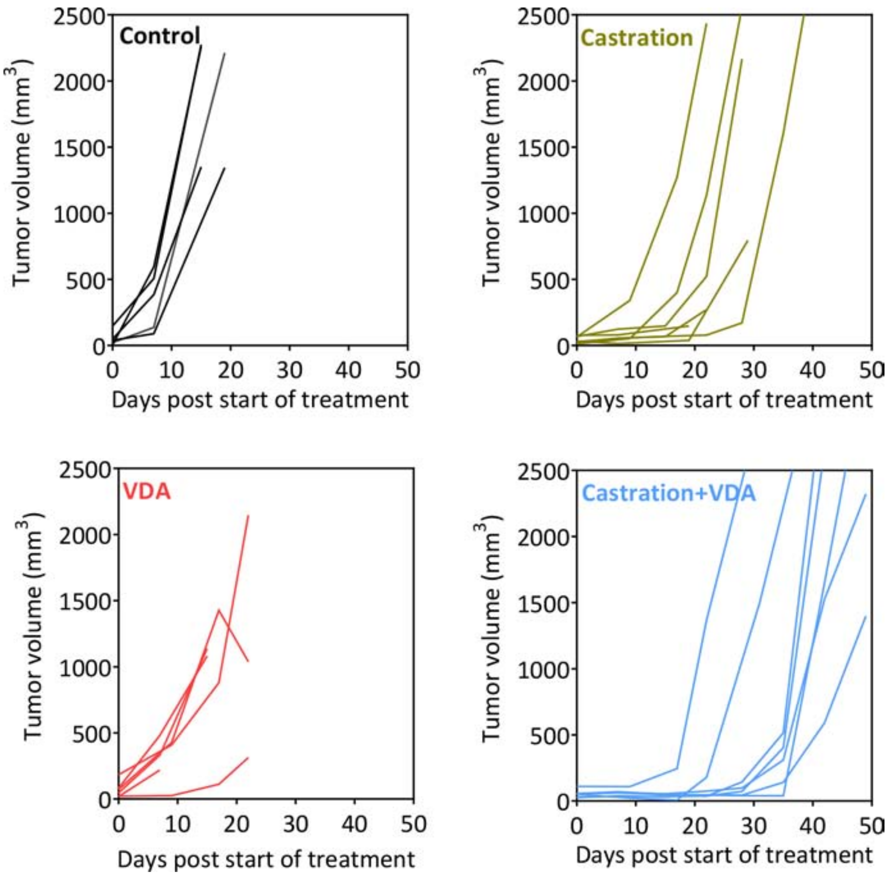

Supplementary Figure S2: MRI-based tumor volume measurements of orthotopic Myc-CaP tumors. Change in volumes of orthotopic Myc-CaP prostate tumors in mice from all 4 experimental groups. Tumor volume was calculated from multislice T2-weighted MR images once a week for the duration of the study.

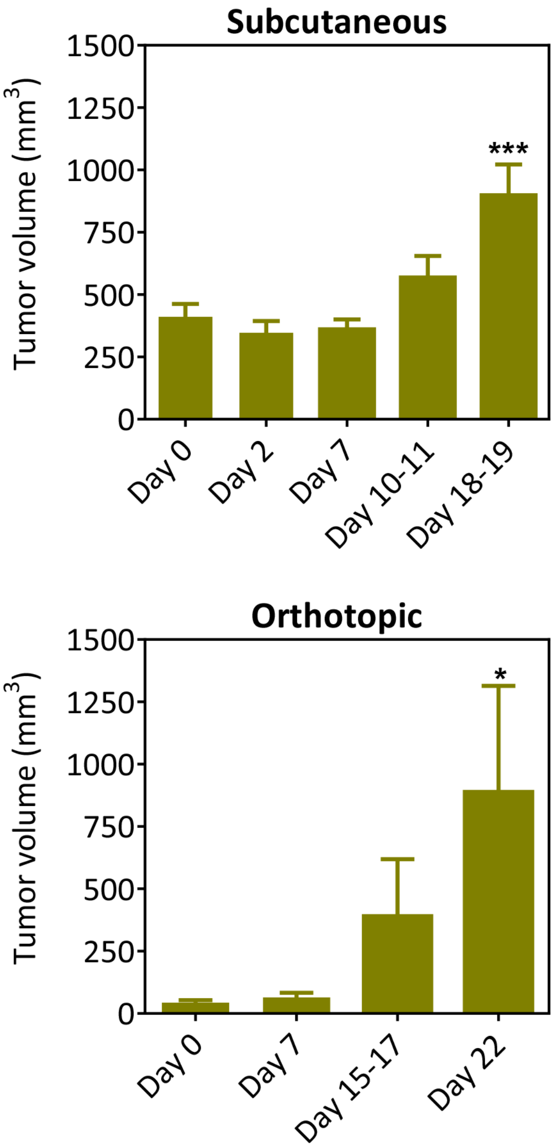

Supplementary Figure S3: Onset of castration recurrent disease in subcutaneous and orthotopic Myc-CaP tumors. Change in tumor volume (days post castration) of subcutaneous and orthotopic Myc-CaP prostate tumors following surgical castration (d0). Physical evidence of tumor growth following castration was seen ~10–11 days in subcutaneous tumors and ~15 days in orthotopic tumors. $*p < 0.05$, $***p < 0.005$ (One-way ANOVA with multiple comparisons).
